# An efficient characterization of submodular spanning tree games

**DOI:** 10.1007/s10107-020-01499-w

**Published:** 2020-04-25

**Authors:** Zhuan Khye Koh, Laura Sanità

**Affiliations:** 1 Department of Mathematics, London School of Economics and Political Science, London, UK; 2 Department of Combinatorics and Optimization, University of Waterloo, Waterloo, Canada; 3 Department of Mathematics and Computer Science, Eindhoven University of Technology, Eindhoven, Netherlands

**Keywords:** 05C05 Trees, 05C57 Games on graphs (graph-theoretic aspects), 91A12 Cooperative games

## Abstract

*Cooperative games* form an important class of problems in game theory, where a key goal is to distribute a value among a set of players who are allowed to cooperate by forming coalitions. An outcome of the game is given by an allocation vector that assigns a value share to each player. A crucial aspect of such games is *submodularity* (or *convexity*). Indeed, convex instances of cooperative games exhibit several nice properties, e.g. regarding the existence and computation of allocations realizing some of the most important solution concepts proposed in the literature. For this reason, a relevant question is whether one can give a polynomial-time characterization of submodular instances, for prominent cooperative games that are in general non-convex. In this paper, we focus on a fundamental and widely studied cooperative game, namely *the spanning tree game*. An efficient recognition of submodular instances of this game was not known so far, and explicitly mentioned as an open question in the literature. We here settle this open problem by giving a polynomial-time characterization of submodular spanning tree games.

## Introduction

*Cooperative games* are among the most studied classes of problems in game theory, with plenty of applications in economics, mathematics, and computer science. In such games, a central question is how to distribute cost (or revenue) among a set of participants, usually called *players*, who are allowed to cooperate. Formally, we are given a set *N* of players, and a characteristic function $\nu : 2^N \rightarrow {\mathbb {R}}$, with $\nu (\emptyset ) = 0$. Here, $\nu (S)$ represents the cost paid (revenue received) by the subset *S* of players if they choose to form a coalition. An outcome of the game is given by an *allocation* $y \in {\mathbb {R}}^N$ such that $\sum _{v \in N} y_v = \nu (N)$, which assigns a cost (revenue) share to each player. Of course, there are a number of criteria for evaluating how “good” an allocation is, such as *stability*, *fairness*, and so on.

Probably the most popular solution concept for cooperative games is the *core*. It is the set of stable outcomes where no subset of players has an incentive to form a coalition to deviate. In a cooperative cost game, this translates naturally to the following constraint: $\sum _{v \in S} y_v \le \nu (S)$, for all $S \subseteq N$. Intuitively, if this constraint is violated for some set *S*, the total cost currently paid by the players in *S* is more than the total cost $\nu (S)$ they would have to pay if they form a coalition—this incentivizes these players to deviate from the current allocation. Besides the core, there are several other crucial solution concepts which have been defined in the literature, e.g. the *Shapley value*, the *nucleolus*, the *kernel*, the *bargaining set*, and the *von Neumann-Morgenstern solution sets* (we refer to [2] for details). Many fundamental questions involving such solution concepts have been investigated in the past few decades: Which cooperative game instances admit an allocation realizing a particular solution concept? Can we efficiently compute it? Can we test whether a given allocation belongs to such sets?

*Submodularity* (or *convexity*) is a crucial property which yields interesting answers to some of the questions above. An instance of a cooperative cost game is called submodular if the characteristic function $\nu$ is submodular, meaning that

* $$\begin{aligned} \forall A, B \subseteq N, \; \nu (A) + \nu (B) \ge \nu (A \cup B) + \nu (A \cap B). \end{aligned}$$

Submodular games exhibit a large number of desirable properties. In particular, (1) the core is always non-empty and an allocation in the core can be computed in polynomial time [13]; (2) testing whether an allocation belongs to the core is equivalent to separating over the extended polymatroid of $\nu$, which can be performed efficiently [8]; (3) computing the nucleolus can be done efficiently [10]; (4) there is a nice “snowballing” effect that arises when the game is played cooperatively, meaning that joining a coalition becomes more attractive as the coalition grows, and so the value of the so-called grand coalition $\nu (N)$ is always reached [13]. We refer to [11, 13] for other interesting properties of submodular games involving other crucial solution concepts. Given these observations, it is not surprising that some researchers have investigated whether it is possible to give an efficient characterization of submodular instances, for prominent cooperative games that are in general non-convex. Such characterizations are known, for example, for the minimum coloring game and the minimum vertex cover game [12], as well as for some communication games [15].

This paper focuses on one of the most fundamental cooperative games, namely the *spanning tree game*. This game was introduced more than 40 years ago [1, 3], and since then it has been widely studied in the literature. To get an intuition about the problem, consider the following setting. A set *N* of clients would like to be connected to a central source *r* which can provide a service to them. The clients wish to build a network connecting them to the source *r*, at minimum cost. An obvious way to solve this problem is to compute a minimum spanning tree connecting $N\cup \left\{ r\right\}$. But how should the clients fairly split the cost of the tree among them? Formally, an instance of the spanning tree game is described by an edge-weighted complete graph $G=(V,E)$ where $V=N\cup \left\{ r\right\}$. The set of players is given by *N*, and the value $\nu (S)$ of the characteristic function is equal to the cost of a minimum spanning tree in the subgraph induced by $S \cup \{r\}$.

Despite being one of the most studied cooperative games, the existence of an efficient characterization of submodularity for the spanning tree game has remained elusive so far. Granot and Huberman [7] proved that spanning tree games are *permutationally convex* (which is a generalization of submodularity). Their result implies that the core is always non-empty for such games, despite being non-convex in general (this was first proven by the same authors in [6]). However, other nice properties of submodular games do not generalize: for general spanning tree games, testing core membership is coNP-hard [4], and computing the nucleolus is NP-hard [5]. Trudeau [14] gave a sufficient condition for an instance of the game to be submodular. An important step forward was made by Kobayashi and Okamoto [9], who gave a characterization of submodularity for instances of the spanning tree game where the edge weights are restricted to take only two values. For general weights, they stated some necessary (but not always sufficient) as well as some sufficient (but not always necessary) conditions for an instance to be submodular. Whether a polynomial-time characterization of submodularity exists for spanning tree games is left as an open question. In fact, they stated twice in their paper:

*“We feel that recognizing a submodular minimum-cost spanning tree game is coNP-complete, but we are still far from proving such a result.”*

**Our results and techniques.** In this paper, we finally settle this open question: we give a polynomial-time characterization of submodular spanning tree games.

Our characterization uses combinatorial techniques and it is based on two main ingredients. The first one, described in Sect. 3, is a generalization of Kobayashi and Okamoto’s result [9]. When the edges can have only two distinct weights, they proved that the only obstruction to submodularity comes from the presence of certain cycles in the graph induced by the cheaper edges. When dealing with more weight values, say $w_1<w_2<\dots <w_k$, things become necessarily more complicated. We can still prove that an obstruction to submodularity is given by certain cycles, which we call *violated*, but (a) our definition of violated cycles is more involved than the one in [9], and (b) we have to look for such cycles not just in one induced graph, but in each graph induced by the edges of weight at most $w_i$, for all $i<k$.

Furthermore, the presence of violated cycles is not anymore the only obstruction to submodularity. Roughly speaking, violated cycles capture how the edges of a certain weight should relate to the cheaper ones, but we still need a condition that takes into account the “magnitude” of distinct weight values, when $k>2$. This leads to the second main ingredient of our characterization, described in Sect. 4. We show that, under the assumption of not having violated cycles, we can identify polynomially many subsets of vertices which could yield the highest possible violation to the submodularity inequality (*). We can then efficiently test the submodularity of our instance by checking whether the inequality (*) is satisfied on this family of subsets of vertices. Combining these two ingredients yields a polynomial-time characterization of submodularity for spanning tree games, as described in Sect. 5.

We conclude our paper with an additional result. As previously mentioned, the authors of [9] gave a necessary condition for submodularity of the spanning tree game. They also stated that they do not know whether their condition can be verified in polynomial time. We answer this question affirmatively in Sect. 6.

## Preliminaries and notation

For a subset $S\subseteq V$, denote $\textsf {mst}(S)$ as the weight of a minimum spanning tree in *G*[*S*], where *G*[*S*] is the subgraph of *G* induced by *S*. For a subset $F\subseteq E$, denote *w*(*F*) as the sum of edge weights in *F*, i.e. $w(F) := \sum _{e\in F}w(e)$. Let *H* be a subgraph of *G*. For a vertex $u\in V$ and an edge $e\in E$, the subgraphs $H{\setminus } u$ and $H{\setminus } e$ are defined as $H{\setminus } u:= H[V(H){\setminus } u]$ and $H{\setminus } e:= (V(H), E(H){\setminus } e)$. Given a vertex $u\in V$, $N_H(u)$ is the neighborhood of *u* in *H*, while $\delta _H(u)$ is the set of edges incident to *u* in *H*. Note that $u\notin N_H(u)$. For any positive integer *k*, [*k*] represents the set $\left\{ 1,2,\dots ,k\right\}$. Given a pair of vertices $u,v\in N$, let ${\mathcal {S}}_{uv}$ denote the family of vertex subsets which contain *r* but not *u* or *v*, i.e. ${\mathcal {S}}_{uv}:=\left\{ S\subseteq V:r\in S \text{ and } u,v\notin S\right\}$. Define the function $f_{uv}:{\mathcal {S}}_{uv}\rightarrow {\mathbb {R}}$ as

$$\begin{aligned} f_{uv}(S) := \textsf {mst}(S\cup u) + \textsf {mst}(S\cup v) - \textsf {mst}(S) - \textsf {mst}(S\cup \left\{ u,v\right\} ). \end{aligned}$$

It is easy to see that the spanning tree game on *G* is submodular if and only if $f_{uv}(S)\ge 0$ for all $u,v\in N$ and $S\in {\mathcal {S}}_{uv}$. Let $w_1<w_2<\dots <w_k$ be the edge weights of *G*. For each $i\in [k]$, define the graph $G_i:=(V,E_i)$ where $E_i:=\left\{ e\in E:w(e)\le w_i\right\}$. Note that $G_k=G$. For a vertex $u\in V$, denote $N_i(u)$ as the neighborhood of *u* in $G_i$. For an edge $uv\in E$, define the neighborhood of *uv* in $G_i$ as

$$\begin{aligned} N_i(uv) := N_i(u) \cap N_i(v). \end{aligned}$$

It represents the set of vertices whose edges to *u* and *v* have weight at most $w_i$. Notice that $u,v\notin N_i(uv)$. We will also need the following graph theory terminology. A *hole* is an induced cycle with at least four vertices. A *diamond* is the complete graph $K_4$ minus one edge. We will refer to the vertices of degree 2 in a diamond as *tips*. Lastly, the following property of minimum spanning trees will be useful to us.

### Lemma 1

Let *T* be a minimum spanning tree of *G*. For every subset $S\subseteq V$, there exists a minimum spanning tree of *G*[*S*] which contains *E*(*T*[*S*]).

### Proof

Let $T_S$ be a minimum spanning tree of *G*[*S*]. We proceed by induction on $\left| E(T[S]){\setminus } E(T_S)\right|$. For the base case, if $\left| E(T[S]){\setminus } E(T_S)\right| =0$, then $T_S$ contains all the edges in *E*(*T*[*S*]). For the inductive step, assume $\left| E(T[S]){\setminus } E(T_S)\right| >0$. Then, there exists an edge $e\in E(T[S])$ where $e\notin E(T_S)$. Adding *e* to $T_S$ creates a cycle *C* in *G*[*S*]. So there exists an edge $f\in E(C)$ which is not an edge of *T*. Pick an appropriate *f* such that when added to *T* creates a cycle containing *e*. Since $T+f-e$ is a spanning tree of *G*, we have $w(f)\ge w(e)$. On the other hand, since $T_S+e-f$ is a spanning tree of *G*[*S*], we also have $w(e)\ge w(f)$. This implies $w(e)=w(f)$, so $T_S+e-f$ is a minimum spanning tree of *G*[*S*]. As $\left| E(T[S]){\setminus } E(T_S+e-f)\right| = \left| E(T[S]){\setminus } E(T_S)\right| -1$, by the induction hypothesis we are done. $\square$

## Violated cycles

In this section, we will prove that a submodular spanning tree game does not contain violated cycles, which will be defined later. First, we need to introduce the concept of *well-covered* cycles.

### Definition 1

Given a cycle *C* and a chord $f=uv$, let $P_1$ and $P_2$ denote the two *u*-*v* paths in *C*. The cycles $P_1+f$ and $P_2+f$ are called the *subcycles of C formed by f*. We say that *f* *covers* *C* if $w(f)\ge w(e)$ for all $e\in E(P_1)$ or for all $e\in E(P_2)$. If *C* is covered by all of its chords, then it is *well-covered*.

**Fig. 1:**
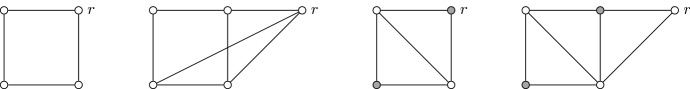
The first two graphs are examples of bad holes, whereas the last two graphs are examples of bad induced diamonds. The tips of the diamonds are shaded. Every edge here has the same weight

Next, we define the following two simple structures (see Fig. 1 for some examples). We then proceed to show that a submodular spanning tree game does not contain either of them.

### Definition 2

A hole is *bad* if at least one of its vertices is not adjacent to *r*. An induced diamond is *bad* if its Hamiltonian cycle is well-covered but at least one of its tips is not adjacent to *r*.

### Lemma 2

If the spanning tree game on *G* is submodular, then there are no (a) bad holes or (b) bad induced diamonds in $G_i$ for any $i<k$.

The lemma will be proved via contrapositive separately for (a) and (b). Before jumping into the details, it is instructive to have an overview of the arguments, as they revolve around the same idea. The proof starts by assuming the existence of a bad hole or a bad induced diamond in $G_i$ for some $i<k$. Then, from this bad structure, a subset of vertices $S\ni r$ and two additional vertices *u*, *v* will be identified such that they violate the submodularity inequality, i.e. $f_{uv}(S)<0$.

### Proof of Lemma 2(a)

Let *C* be a bad hole in $G_i$ for some $i<k$. Consider the following two cases.

**Fig. 2:**
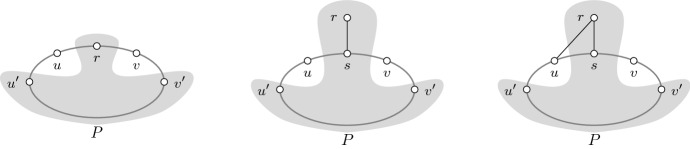
An example of the subgraph $G_i[S\cup \left\{ u,v\right\} ]$ in Case 1 and Subcases 2.1–2.2 respectively. The shaded region represents the set *S*

*Case 1:* *C* *contains* *r*. Let *u*, *v* be the vertices adjacent to *r* in *C*. Define the set $S:=V(C){\setminus }\left\{ u,v\right\}$. To prove that the instance is not submodular, it suffices to show that $f_{uv}(S)<0$. Let *P* be the path obtained by deleting *r*, *u*, *v* from *C*. Let $u',v'$ be the endpoints of *P* where $uu',vv'\in E(C)$ (see Fig. 2). Note that $u'=v'$ if *P* is a singleton. It is easy to see that

$$\begin{aligned} \textsf {mst}(S)&\ge w(P) + w_{i+1}, \\ \textsf {mst}(S\cup u)&= w(P) + w(ru) + w(uu'), \\ \textsf {mst}(S\cup v)&= w(P) + w(rv) + w(vv'). \end{aligned}$$

Next, deleting the most expensive edge from *C* creates a minimum spanning tree of $G[S\cup \left\{ u,v\right\} ]$. Since this edge has weight at most $w_i$, we obtain

$$\begin{aligned} \textsf {mst}(S\cup \left\{ u,v\right\} ) \ge w(P) + w(ru) + w(uu') + w(rv) + w(vv') - w_i. \end{aligned}$$

Combining the equations and inequalities above yields

$$\begin{aligned} f_{uv}(S) = \textsf {mst}(S\cup u) + \textsf {mst}(S\cup v) - \textsf {mst}(S) - \textsf {mst}(S\cup \left\{ u,v\right\} ) \le w_i - w_{i+1} < 0. \end{aligned}$$

*Case 2: C does not contain r.* We first show that if *r* is adjacent in $G_i$ to two non-adjacent vertices of *C*, then we are done. Since *C* is a bad hole in $G_i$, there exists a vertex $t\in V(C)$ such that $rt\notin E_i$. Starting from *t*, traverse the hole *C* in both directions until we encounter the first vertices *p* and *q* such that $rp,rq\in E_i$ respectively. Since *r* is adjacent to two non-adjacent vertices of *C*, we have $p\ne q$ and $pq\notin E(C)$. Let *Q* be the *p*-*q* path in *C* which contains *t*. Then, by our choice of *p* and *q*, the cycle $Q+rp+rq$ is a bad hole in $G_i$ which contains *r*. It follows that the instance is not submodular by Case 1.

Thus, we may assume that *r* can only be adjacent to adjacent vertices of *C*. Pick a vertex $s\in V(C)$ with the cheapest edge to *r*, i.e. $w(rs)\le w(rx)$ for all $x\in V(C)$. Let *u*, *v* be the vertices adjacent to *s* in *C*. Define the set $S:=(V(C)\cup r){\setminus }\left\{ u,v\right\}$. As with the previous case, it suffices to show that $f_{uv}(S)<0$. Let *P* be the path obtained by deleting *s*, *u*, *v* from *C*. Let $u',v'$ be the endpoints of *P* where $uu',vv'\in E(C)$ (see Fig. 2). We are left with the following two subcases.

*Subcase 2.1:* *r* *is adjacent to at most one vertex of* *C*. Since *s* was chosen to be the vertex in *C* with the cheapest edge to *r*, we have

$$\begin{aligned} \textsf {mst}(S)&\ge w(P) + w(rs) + w_{i+1}, \\ \textsf {mst}(S\cup u)&= w(P) + w(rs) + w(su) + w(uu'), \\ \textsf {mst}(S\cup v)&= w(P) + w(rs) + w(sv) + w(vv'). \end{aligned}$$

Next, deleting the most expensive edge from *C* and adding *rs* creates a minimum spanning tree of $G[S\cup \left\{ u,v\right\} ]$. Since the deleted edge has weight at most $w_i$, we obtain

$$\begin{aligned} \textsf {mst}(S\cup \left\{ u,v\right\} ) \ge w(P) + w(rs) + w(su) + w(uu') + w(sv) + w(vv') - w_i. \end{aligned}$$

Combining the equations and inequalities above yields

$$\begin{aligned} f_{uv}(S) = \textsf {mst}(S\cup u) + \textsf {mst}(S\cup v) - \textsf {mst}(S) - \textsf {mst}(S\cup \left\{ u,v\right\} ) \le w_i - w_{i+1} < 0. \end{aligned}$$

*Subcase 2.2:* *r* *is adjacent to two vertices of* *C*. Since we assumed that *r* can only be adjacent to adjacent vertices of *C*, it follows that *r* is adjacent to *s* and either *u* or *v* in $G_i$. Without loss of generality, suppose that $rs,ru\in E_i$. Since *s* was chosen to be the vertex in *C* with the cheapest edge to *r*, we have

$$\begin{aligned} \textsf {mst}(S)&\ge w(P) + w(rs) + w_{i+1}, \\ \textsf {mst}(S\cup u)&= w(P) + w(rs) + \min \left\{ w(ru),w(su)\right\} + w(uu'), \\ \textsf {mst}(S\cup v)&= w(P) + w(rs) + w(sv) + w(vv'). \end{aligned}$$

Next, deleting the most expensive edge from the triangle $\left\{ rs,ru,su\right\}$ does not increase the weight of a minimum spanning tree in $G[S\cup \left\{ u,v\right\} ]$. In fact, a minimum spanning tree can be obtained by deleting one more edge from $G_i[S\cup \left\{ u,v\right\} ]$. So,

$$\begin{aligned} \textsf {mst}(S\cup \left\{ u,v\right\} )\ge & {} w(P) + w(rs) + \min \left\{ w(ru),w(su)\right\} + w(uu') \\&+ w(sv) + w(vv') - w_i. \end{aligned}$$

Combining the equations and inequalities above yields

$$\begin{aligned} f_{uv}(S) = \textsf {mst}(S\cup u) + \textsf {mst}(S\cup v) - \textsf {mst}(S) - \textsf {mst}(S\cup \left\{ u,v\right\} ) \le w_i - w_{i+1} < 0. \end{aligned}$$

$$\square $$

### Proof of Lemma 2(b)

Let *D* be a bad induced diamond in $G_i$ for some $i<k$. Consider the following two cases.

**Fig. 3:**
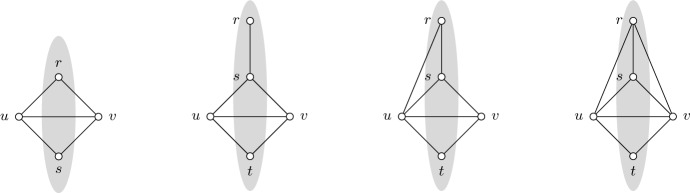
An example of the subgraph $G_i[S\cup \left\{ u,v\right\} ]$ in Case 1 and Subcases 2.1–2.3 respectively. The shaded region represents the set *S*. For Subcase 2.1, the picture assumes that $w(rs)< w(ru)$

*Case 1:* *D* *contains* *r*. Observe that *r* is a tip of *D*. Let *s* be the other tip and *u*, *v* be the non-tip vertices of *D*. Define the set $S:=\left\{ r,s\right\}$ (see Fig. 3). To prove that the instance is not submodular, it suffices to show that $f_{uv}(S)<0$. It is easy to see that

$$\begin{aligned} \textsf {mst}(S)&\ge w_{i+1}, \\ \textsf {mst}(S\cup u)&= w(ru) + w(su), \\ \textsf {mst}(S\cup v)&= w(rv) + w(sv). \end{aligned}$$

Since the Hamiltonian cycle of *D* is well-covered, its chord *uv* can be deleted without increasing the weight of a minimum spanning tree in $G[S\cup \left\{ u,v\right\} ]$. We are now left with the Hamiltonian cycle of *D*, so a minimum spanning tree can be obtained by removing the most expensive edge. This gives

$$\begin{aligned} \textsf {mst}(S\cup \left\{ u,v\right\} ) \ge w(ru) + w(su) + w(rv) + w(sv) - w_i. \end{aligned}$$

Then, combining the equations and inequalities above yields

$$\begin{aligned} f_{uv}(S) = \textsf {mst}(S\cup u) + \textsf {mst}(S\cup v) - \textsf {mst}(S) - \textsf {mst}(S\cup \left\{ u,v\right\} ) \le w_i - w_{i+1} < 0. \end{aligned}$$

*Case 2: D does not contain r.* Let *s* and *t* be the tips of *D* where $w(rs)\le w(rt)$. Note that $rt\notin E_i$ because *D* is a bad induced diamond in $G_i$. Let *u* and *v* be the non-tip vertices of *D* where $w(ru)\le w(rv)$. Define the set $S:=\left\{ r,s,t\right\}$ (see Fig. 3). As with the previous case, it suffices to show that $f_{uv}(S)<0$. Consider the following three subcases.

*Subcase 2.1:* *r* *is adjacent to at most one vertex of* *D*. Note that $rv\notin E_i$. It is also easy to see that

$$\begin{aligned} \textsf {mst}(S)&\ge w(rs) + w_{i+1}, \\ \textsf {mst}(S\cup u)&= \min \left\{ w(rs),w(ru)\right\} + w(su) + w(tu), \\ \textsf {mst}(S\cup v)&= \min \left\{ w(rs),w(rv)\right\} + w(sv) + w(tv). \end{aligned}$$

Next, observe that we can delete *uv* and the most expensive edge in the Hamiltonian cycle of *D* without increasing the weight of a minimum spanning tree in $G[S\cup \left\{ u,v\right\} ]$. Therefore,

$$\begin{aligned} \textsf {mst}(S\cup \left\{ u,v\right\} ) \ge \min \left\{ w(rs),w(ru)\right\} + w(su) + w(tu) + w(sv) + w(tv) - w_i. \end{aligned}$$

Then, combining the equations and inequalities above yields

$$\begin{aligned} f_{uv}(S) = \textsf {mst}(S\cup u) + \textsf {mst}(S\cup v) - \textsf {mst}(S) - \textsf {mst}(S\cup \left\{ u,v\right\} ) \le w_i - w_{i+1} < 0. \end{aligned}$$

*Subcase 2.2:* *r* *is adjacent to two vertices of* *D*. We claim that if $ru,rv\in E_i$, then we are done. Note that this implies $rs,rt\notin E_i$. So, if $w(uv)\ge \max \left\{ w(su),w(sv)\right\}$, then $G[\left\{ r,s,u,v\right\} ]$ is a bad induced diamond in $G_i$. Otherwise, $w(uv)\ge \max \left\{ w(tu),w(tv)\right\}$ because $D{\setminus } uv$ is a well-covered cycle in $G_i$. Hence, $G[\left\{ r,t,u,v\right\} ]$ is a bad induced diamond in $G_i$. Since both diamonds contain *r*, the instance is not submodular by Case 1. Thus, we may assume that $rs,ru\in E_i$. Additionally, we may assume that $w(su)<\max \left\{ w(rs),w(ru)\right\}$. Otherwise, $G[\left\{ r,s,u,v\right\} ]$ is a bad induced diamond in $G_i$, and we are done again by Case 1. With these two assumptions, it is then easy to see that

$$\begin{aligned} \textsf {mst}(S)&\ge w(rs) + w_{i+1}, \\ \textsf {mst}(S\cup u)&= \min \left\{ w(rs),w(ru)\right\} + w(su) + w(tu), \\ \textsf {mst}(S\cup v)&= w(rs) + w(sv) + w(tv). \end{aligned}$$

Since ${{\,\mathrm{arg max}\,}}\left\{ w(rs),w(ru)\right\}$ is the most expensive edge in the cycle $\left\{ rs,ru,su\right\}$ by our assumption, it can be deleted without increasing the weight of a minimum spanning tree in $G[S\cup \left\{ u,v\right\} ]$. By a similar reasoning, *uv* and the most expensive edge in the Hamiltonian cycle of *D* can also be deleted. Therefore,

$$\begin{aligned} \textsf {mst}(S\cup \left\{ u,v\right\} )&\ge \min \left\{ w(rs),w(ru)\right\} + w(su) + w(tu) + w(sv) + w(tv) - w_i. \end{aligned}$$

Finally, combining the equations and inequalities above yields

$$\begin{aligned} f_{uv}(S) = \textsf {mst}(S\cup u) + \textsf {mst}(S\cup v) - \textsf {mst}(S) - \textsf {mst}(S\cup \left\{ u,v\right\} ) \le w_i - w_{i+1} < 0. \end{aligned}$$

*Subcase 2.3:* *r* *is adjacent to three vertices of* *D*. Let $w(rv)=w_j$ for some $j\le i$, and consider the induced diamond $G[\left\{ r,t,u,v\right\} ]$. If its Hamiltonian cycle is well-covered, then we are done by Case 1 because $rt\notin E_i$. So we may assume that $\max \left\{ w(su),w(sv)\right\} \le w(uv) < w(rv)$. Additionally, we may assume that $w(su)<\max \left\{ w(rs),w(ru)\right\}$. Otherwise, from the previous assumption, the five edges $\left\{ rs,ru,su,sv,uv\right\}$ have strictly smaller weights than $w(rv)=w_j$. Thus, $G[\left\{ r,s,u,v\right\} ]$ is a bad induced diamond in $G_{j-1}$, and we are done again by Case 1. With these two assumptions, it is then easy to see that

$$\begin{aligned} \textsf {mst}(S)&\ge w(rs) + w_{i+1}, \\ \textsf {mst}(S\cup u)&= \min \left\{ w(rs),w(ru)\right\} + w(su) + w(tu), \\ \textsf {mst}(S\cup v)&= \min \left\{ w(rs),w(rv)\right\} + w(sv) + w(tv). \end{aligned}$$

Since *rv* is the most expensive edge in the cycle $\left\{ ru,rv,su,sv\right\}$ by our assumption, it can be deleted without increasing the weight of a minimum spanning tree in $G[S\cup \left\{ u,v\right\} ]$. By a similar reasoning, ${{\,\mathrm{arg max}\,}}\left\{ rs,ru\right\}$, *uv* and the most expensive edge in the Hamiltonian cycle of *D* can also be deleted. Hence,

$$\begin{aligned} \textsf {mst}(S\cup \left\{ u,v\right\} ) \ge \min \left\{ w(rs),w(ru)\right\} + w(su) + w(tu) + w(sv) + w(tv) - w_i. \end{aligned}$$

Finally, combining the equations and inequalities above yields

$$\begin{aligned} f_{uv}(S) = \textsf {mst}(S\cup u) + \textsf {mst}(S\cup v) - \textsf {mst}(S) - \textsf {mst}(S\cup \left\{ u,v\right\} ) \le w_i - w_{i+1} < 0. \end{aligned}$$

$$\square $$

We are now ready to define the main object of study in this section.

### Definition 3

A *violated* cycle is a well-covered cycle which contains at least one pair of non-adjacent vertices $u,v\in V$ and at least one vertex $w\in N$ not adjacent to *r*.

In the definition above, *w* can be equal to *u* or *v*. For example, if $r\in V(C)$, then the pair $\left\{ r,w\right\}$ already certifies that *C* is a violated cycle because it can be used to satisfy both conditions. Observe that bad holes and Hamiltonian cycles of bad induced diamonds are examples of violated cycles (we consider a hole to be well-covered). The next lemma extends the scope of Lemma 2 to include violated cycles. When $k=2$, this coincides with the condition given by Kobayashi and Okamoto [9] because every cycle in $G_1$ is well-covered.

### Lemma 3

If the spanning tree game on *G* is submodular, then there are no violated cycles in $G_i$ for any $i<k$.

### Proof

We will prove the contrapositive. Let *j* be the smallest integer such that $G_j$ contains a violated cycle. By our choice of *j*, there are no violated cycles in $G_i$ for all $i<j$. Let *C* be a smallest violated cycle in $G_j$. Then, $\max _{e\in E(C)}w(e)=w_j$. We first prove the following claim.

### Claim 1

For any chord *f*, the subcycles of *C* formed by *f* are well-covered.

### Proof

**Fig. 4:**
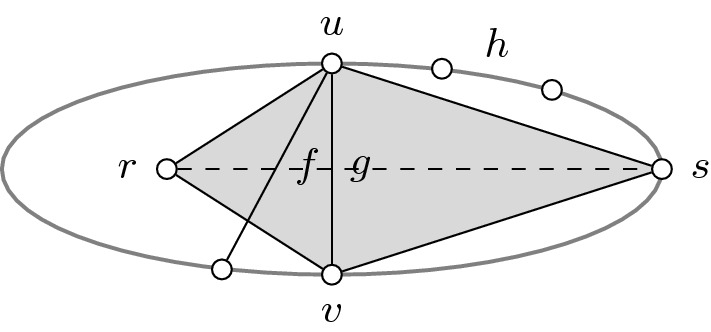
The ellipse represents the violated cycle *C* in Claim 1. The shaded region highlights the smaller violated cycle *D*. The dashed edge indicates $rs\notin E_j$

Let $C_1$ and $C_2$ denote the subcycles of *C* formed by *f*. For the purpose of contradiction, suppose that $C_2$ is not well-covered. Let $g=uv$ be the cheapest chord in $C_2$ such that $w(g)<w(f)$ and $w(g)<w(h)$ for some edge $h\in E(C_2)$, where *f* and *h* lie in different subcycles of $C_2$ formed by *g* (see Fig. 4 for an example). This chord exists because *C* is well-covered but $C_2$ is not. Consider the subcycles $C_3$ and $C_4$ of *C* formed by *g*, where *f* is a chord of the former. Observe that any chord of $C_3$ covers $C_3$ because *C* is well-covered and $w(g)<w(h)$. So, the subcycle $C_3$ is well-covered. In addition, the subcycle $C_4$ is well-covered because *g* was chosen to be the cheapest chord preventing $C_2$ from being well-covered.

Let $w(g)=w_\ell$ for some $\ell <j$. We claim that the subcycle $C_3$ is present in $G_\ell$. To see this, recall that *C* is well-covered and $w(g)<w(h)$. Hence, it follows that $w(g)\ge w(e)$ for all $e\in E(C_3)$, which proves the claim. On the other hand, notice that the chord *f* is absent from $G_\ell$ because $w(f)>w(g)$. Since the subcycle $C_3$ is well-covered, its vertices are adjacent to *r* in $G_\ell$ because there are no violated cycles in $G_\ell$. Next, recall that *C* is a violated cycle in $G_j$, so there exists a vertex $s\in V(C_4){\setminus } V(C_3)$ such that $rs\notin E_j$. Since the subcycle $C_4$ is well-covered, its vertices are pairwise adjacent in $G_j$, as otherwise it is a smaller violated cycle than *C*. Note that this also implies $r\notin V(C_4)$. Thus, we have $ru,rv\in E_\ell$ and $su,sv\in E_j$.

Now, consider the 4-cycle *D* defined by $E(D):=\left\{ ru,rv,su,sv\right\}$. It is well-covered because $w(g)=w_\ell$ and $ru,rv\in E_\ell$. Since $rs\notin E_j$, it is also a violated cycle in $G_j$. However, it is smaller than *C*. Indeed, *C* has at least 5 vertices because *f* is a chord in $C_3$ while *g* is a chord in $C_2$. We have arrived at a contradiction. $\square$

Our goal is to show the existence of a bad hole or a bad induced diamond in $G_j$. Then, we can invoke Lemma 2 to conclude that the game is not submodular. We may assume that *C* has a chord, otherwise it is trivially a bad hole. First, consider the case when $r\in V(C)$. Let $s\in V(C)$ where $rs\notin E_j$. For any chord *f* in *C*, observe that *r* and *s* lie in different subcycles of *C* formed by *f*. This is because the subcycles are well-covered by the previous claim, so the one which contains both *r* and *s* will contradict the minimality of *C*. Now, let *g* be a chord of *C*. Let $C_r$ and $C_s$ denote the subcycles of *C* formed by *g* where $r\in V(C_r)$ and $s\in V(C_s)$. Observe that the vertices of $C_r$ are adjacent to *r* due to the minimality of *C*. Thus, $C_r$ is a triangle. Otherwise, there is a chord in $C_r$ incident to *r*, and it forms a subcycle of *C* which contains both *r* and *s*. On the other hand, the vertices of $C_s$ are pairwise adjacent due to the minimality of *C*. Hence, $C_s$ is also a triangle. Otherwise, there exists a chord in $C_s$ incident to *s*, and it forms a subcycle of *C* which contains both *r* and *s*. Therefore, *C* is a bad induced diamond in $G_j$.

Next, consider the case when $r\notin V(C)$. From this point forward, we may assume that every smallest violated cycle in $G_j$ does not contain *r*. Otherwise, we are back in the first case again. With this additional assumption, non-adjacency within *C* implies non-adjacency with *r*, as shown by the following claim.

### Claim 2

For any pair of vertices $u,v\in V(C)$ such that $uv\notin E_j$, we have $ru\notin E_j$ or $rv\notin E_j$.

### Proof

**Fig. 5:**
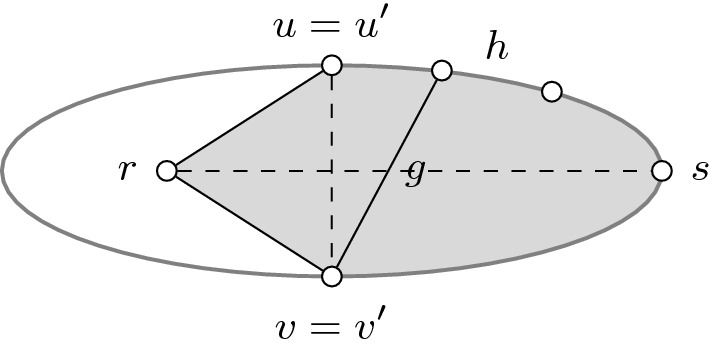
The ellipse represents the violated cycle *C* in Claim 2. The shaded region highlights the violated cycle *D*. The dashed edges indicate non-adjacency in $G_j$. In this example, $u=u'$ and $v=v'$

For the purpose of contradiction, suppose that $ru,rv\in E_j$. Let $s\in V(C)$ such that $rs\notin E_j$. Let $P_{su}$ and $P_{sv}$ denote the edge-disjoint *s*-*u* and *s*-*v* paths in *C* respectively. Let $u'$ and $v'$ be the closest vertex to *s* on $P_{su}$ and $P_{sv}$ respectively such that $ru',rv'\in E_j$ (see Fig. 5 for an example). Without loss of generality, let $w(ru')\ge w(rv')$. Denote $P_{su'}$ and $P_{sv'}$ as the *s*-$u'$ and *s*-$v'$ subpaths of $P_{su}$ and $P_{sv}$ respectively. Now, consider the cycle $D:=P_{su'}+P_{sv'}+ru'+rv'$. Observe that it contains *r* and is no bigger than *C*. Furthermore, it does not contain a chord incident to *r* by our choice of $u'$ and $v'$. To arrive at a contradiction, it is left to show that *D* is well-covered, as this would imply *D* is violated. Suppose for a contradiction, that *D* is not well-covered. Then, there exists a chord *g* in *D* such that $w(g)<w(ru')$ and $w(g)<w(h)$ for some $h\in E(D)$, where $ru'$ and *h* lie in different subcycles of *D* formed by *g*. This chord exists because *C* is well-covered but *D* is not. Let $C_1$ and $C_2$ denote the subcycles of *C* formed by *g*, where $h\in E(C_2)$. Note that any chord of $C_1$ covers $C_1$ because *C* is well-covered and $w(g)<w(h).$ Hence, the subcycle $C_1$ is well-covered. Moreover, we also have $w(g)\ge w(e)$ for all $e\in E(C_1)$ because *C* is well-covered and $w(g)<w(h)$. Let $w(g)=w_\ell$ for some $\ell <j$. Then, $C_1$ is still present in $G_\ell$ but $ru'$ is not. Since $C_1$ also contains *u* and *v* but $uv\notin E_\ell$ because $uv\notin E_j$, it is a violated cycle in $G_\ell$. However, this is a contradiction because there are no violated cycles in $G_\ell$. $\square$

The remaining proof proceeds in a similar fashion to the first case. Let $u,v\in V(C)$ be such that $uv\notin E_j$. By the claim above, we know that $ru\notin E_j$ or $rv\notin E_j$. For any chord *f* in *C*, observe that *u* and *v* lie in different subcycles of *C* formed by *f*. This is because the subcycles are well-covered by Claim 1, so the one which contains both *u* and *v* will contradict the minimality of *C*. Now, let *g* be a chord of *C*. Let $C_u$ and $C_v$ denote the subcycles of *C* formed by *g* where $u\in V(C_u)$ and $v\in V(C_v)$. The vertices of $C_u$ are pairwise adjacent due to the minimality of *C*. Thus, $C_u$ is a triangle. Otherwise, there exists a chord in $C_u$ incident to *u*, and it forms a subcycle of *C* which contains both *u* and *v*. By an analogous argument, $C_v$ is also a triangle. Therefore, *C* is a bad induced diamond in $G_j$. $\square$

Notice that we have proven something stronger. Namely, if $G_j$ contains a violated cycle, then there exists an index $i\le j$ such that $G_i$ contains a bad hole or a bad induced diamond. Moreover, as mentioned earlier, bad holes and Hamiltonian cycles of bad induced diamonds are violated cycles themselves. Thus, we obtain the following corollary.

### Corollary 1

There are no bad holes or bad induced diamonds in $G_i$ for any $i<k$ if and only if there are no violated cycles in $G_i$ for any $i<k$.

## Candidate edges and expensive neighborhood

In the previous section, we showed that violated cycles are an obstruction to submodularity. In light of this fact, we now focus on graphs *G* whose subgraphs $G_i$ do not contain violated cycles. The goal of this section is to study the behaviour of $f_{uv}$ on these graphs. As a first step, the following lemma sheds light on how a minimum spanning tree changes under vertex removal.

### Lemma 4

Assume that there are no violated cycles in $G_i$ for any $i<k$. Let *T* be a minimum spanning tree of *G*[*S*] where $r\in S\subseteq V$. For any $s\ne r$, there exists a minimum spanning tree of $G[S{\setminus } s]$ which contains $E(T{\setminus } s)$ and additionally, only uses edges from $G[N_T(s)\cup r]$.

### Proof

Pick a vertex $s\in S{\setminus } r$. By Lemma 1, there exists a minimum spanning tree of $G[S{\setminus } s]$ which contains $E(T{\setminus } s)$. Let $T'$ be such a tree which uses the most edges from $G[N_T(s)\cup r]$. We will show that $T'$ is our desired tree. For the purpose of contradiction, suppose $T'$ has an edge *uv* such that $uv\notin E(T)$ and $u\notin N_T(s)\cup r$. Note that *u* and *v* lie in different components of $T{\setminus } s$. Let $P_{su}$ and $P_{sv}$ denote the unique *s*-*u* and *s*-*v* paths in *T* respectively. We claim that $C:=P_{su} + P_{sv} + uv$ is a well-covered cycle in $G_i$, where $w_i=w(uv)$. First, observe that *uv* is the most expensive edge in *C* because $P_{su} + P_{sv}$ is part of the minimum spanning tree *T*. Hence, the cycle *C* is present in $G_i$. Let $f=xy$ be any chord of *C* in $G_i$, and denote $P_{xy}$ as the unique *x*-*y* path in *T*. Then, $P_{xy} + f$ is a subcycle of *C* formed by *f*. Moreover, we have $w(f)\ge w(e)$ for all $e\in E(P_{xy})$ because $P_{xy}$ is part of the minimum spanning tree *T*. Hence, the chord *f* covers *C*, which proves the claim.

Let $u'$ and $v'$ be the vertices adjacent to *s* in $P_{su}$ and $P_{sv}$ respectively. By our choice of $T'$, we have $w(u'v')>w(uv)=w_i$, which implies that $u'$ and $v'$ are not adjacent in $G_i$. Since there are no violated cycles in $G_i$, it follows that the vertices of *C* are adjacent to *r* in $G_i$. However, note that adding $ru'$ or $rv'$ to $T'$ creates a fundamental cycle which uses the edge *uv*. Swapping it with *uv* creates another minimum spanning tree of $G[S{\setminus } s]$ which contains $E(T{\setminus } s)$ and uses more edges from $G[N_T(s)\cup r]$. We have arrived at a contradiction. $\square$

Given a pair of vertices $u,v\in N$ where $w(uv)=w_i$, the following definition distinguishes the neighbours of *u*, *v* in *G* from the neighbours of *u*, *v* in $G_i$.

### Definition 4

For an edge $uv\in E$, if $w(uv)=w_i$, the *expensive neighborhood* of *uv* is defined as

$$\begin{aligned} {\hat{N}}(uv) := N_k(uv){\setminus } N_i(uv). \end{aligned}$$

In other words, the expensive neighborhood of an edge *uv* is the set of vertices $s\notin \left\{ u,v\right\}$ such that $\max \left\{ w(su),w(sv)\right\} >w(uv)$. It turns out that the function $f_{uv}$ always returns zero when evaluated on a set which does not lie entirely in the expensive neighborhood of *uv*.

### Lemma 5

Assume that there are no violated cycles in $G_i$ for any $i<k$. Let $u,v\in N$ and $S\in {\mathcal {S}}_{uv}$. If $S\not \subseteq {\hat{N}}(uv)$, then $f_{uv}(S)=0$.

### Proof

Let *T* be a minimum spanning tree of $G[S\cup \left\{ u,v\right\} ]$. First, we show that we can assume $uv\notin E(T)$. Since $S\not \subseteq {\hat{N}}(uv)$, there exists a vertex $s\in S$ such that $\max \left\{ w(su),w(sv)\right\} \le w(uv)$. If $uv\in E(T)$, then by rooting *T* at *s*, *u* is either a child or a parent of *v*. Adding *su* to *T* in the former and *sv* in the latter creates a fundamental cycle which contains *uv*. Thus, we can replace *uv* with this new edge to obtain the desired tree.

Since there are no violated cycles in $G_i$ for any $i<k$, by Lemma 4, there exists a minimum spanning tree $T'$ of $G[S\cup v]$ which contains $E(T{\setminus } u)$ and additionally, only uses edges from $G[N_T(u)\cup r]$. Recall that we assumed $uv\notin E(T)$, or equivalently, $v\notin N_T(u)$. Therefore, the neighborhood of *v* is identical in both trees, i.e. $N_T(v)=N_{T'}(v)$.

**Fig. 6:**
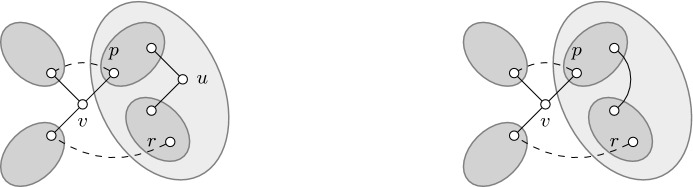
The left image depicts an example of the minimum spanning tree *T* in $G[S\cup \left\{ u,v\right\} ]$. The right image depicts an example of the minimum spanning tree $T'$ in $G[S\cup v]$. The solid edges belong to the trees while dashed edges belong to the edge set *F*

Consider the forest $T{\setminus } v$. Let $p\in N_T(v)$ such that *p* and *r* lie in the same component of $T{\setminus } v$ (see Fig. 6 for an example). Note that $p = r$ if $r\in N_T(v)$. We claim that *p* and *r* also lie in the same component of the forest $T'{\setminus } v$. We may assume that $p\ne r$, as otherwise the claim is trivially true. Moreover, we may assume that *u* lies on the unique *p*-*r* path in *T*. Otherwise, we are done because the same path is present in $T'{\setminus } v$. Let $C_r$ denote the component of $T{\setminus } v$ which contains *p*, *r* and *u*. By Lemma 4, the endpoints of every edge in $E(T'){\setminus } E(T{\setminus } u)$ lie in $C_r$. This proves the claim.

Using Lemma 4, we can construct a minimum spanning tree of $G[S\cup u]$ by deleting *v* from *T* and adding a set *F* of edges from $G[N_T(v)\cup r]$. Note that $pr\notin F$ as *p* and *r* lie in the same component of $T{\setminus } v$. Since *p* and *r* also lie in the same component of $T'{\setminus } v$ and $N_T(v)=N_{T'}(v)$, deleting *v* from $T'$ and adding the same set *F* of edges produces a minimum spanning tree of *G*[*S*]. Thus, we get

$$\begin{aligned} f_{uv}(S)&= \textsf {mst}(S\cup u) + \textsf {mst}(S\cup v) - \textsf {mst}(S) - \textsf {mst}(S\cup \left\{ u,v\right\} ) \\&= \Big (\textsf {mst}(S\cup u) - w(E(T))\Big ) - \Big (\textsf {mst}(S) - w(E(T'))\Big ) \\&= \Big (w(F) - w(\delta _T(v))\Big ) - \Big (w(F) - w(\delta _{T'}(v))\Big ) = 0 \end{aligned}$$

as desired. $$\square $$

We can now focus solely on vertex sets which lie entirely in the expensive neighborhood of *uv*. Observe that if $r\notin {\hat{N}}(uv)$, then $S\not \subseteq {\hat{N}}(uv)$ for all $S\in {\mathcal {S}}_{uv}$. Thus, we do not have to check these edges *uv* as $f_{uv}(S)=0$ for all $S\in {\mathcal {S}}_{uv}$ by the previous lemma. This motivates the following definition.

### Definition 5

An edge $uv\in E$ is called a *candidate edge* if $r\in {\hat{N}}(uv)$.

With a mild assumption, we can show that the function $f_{uv}$ is inclusion-wise nonincreasing in the expensive neighborhood of *uv*.

### Lemma 6

Assume that there are no violated cycles in $G_i$ for any $i<k$. Let *uv* be a candidate edge and $S\in {\mathcal {S}}_{uv}$ such that $S\subseteq {\hat{N}}(uv)$. If $f_{xy}({\hat{N}}(xy)) \ge 0$ for every candidate edge *xy*, then $f_{uv}(S)\le f_{uv}(S{\setminus } s)$ for any $s\ne r$.

### Proof

Pick a vertex $s\in S{\setminus } r$. Without loss of generality, assume $w(su)\ge w(sv)$. Then, $w(su)>w(uv)$ because $s \in {\hat{N}}(uv)$. However, these two inequalities also imply that $v\notin {\hat{N}}(su)$. It follows that the set $(S{\setminus } s)\cup v$ is not contained in the expensive neighborhood of *su*. By Lemma 5,

$$\begin{aligned} 0 = f_{su}((S{\setminus } s)\cup v)= & {} \textsf {mst}(S\cup v) + \textsf {mst}((S{\setminus } s)\cup \left\{ u,v\right\} ) \\&- \textsf {mst}((S{\setminus } s)\cup v) - \textsf {mst}(S\cup \left\{ u,v\right\} ). \end{aligned}$$

Rearranging yields

1 $$\begin{aligned} \textsf {mst}(S\cup v) - \textsf {mst}(S\cup \left\{ u,v\right\} ) = \textsf {mst}((S{\setminus } s)\cup v) - \textsf {mst}((S{\setminus } s)\cup \left\{ u,v\right\} ). \end{aligned}$$

Since *uv* is a candidate edge, let $w(uv)=w_i$ for some $i<k$. We will proceed by induction on *i*. For the base case $i=k-1$, we have $w_{k-1} = w(uv)<w(su)=w_k$. Since ${\hat{N}}(su)=\emptyset$, the set $S{\setminus } s$ is not contained in the expensive neighborhood of *su* because $r\in S{\setminus } s$. By Lemma 5,

$$\begin{aligned} 0 = f_{su}(S{\setminus } s) = \textsf {mst}(S) + \textsf {mst}((S{\setminus } s)\cup u) - \textsf {mst}(S{\setminus } s) - \textsf {mst}(S\cup u). \end{aligned}$$

Rearranging yields

2 $$\begin{aligned} \textsf {mst}(S\cup u) - \textsf {mst}(S) = \textsf {mst}((S{\setminus } s)\cup u) - \textsf {mst}(S{\setminus } s). \end{aligned}$$

Adding (1) and (2) gives $f_{uv}(S)=f_{uv}(S{\setminus } s)$. Now, suppose the lemma is true for all $i\ge j$ for some $j<k$. For the inductive step, let $w(uv)=w_{j-1}$. We may assume that $S{\setminus } s \subseteq {\hat{N}}(su)$, as otherwise we obtain equality again. This implies that *su* is a candidate edge because $r\in S{\setminus } s$. Since $w(su)>w(uv)=w_{j-1}$, we get

$$\begin{aligned} 0\le & {} f_{su}({\hat{N}}(su)) \le f_{su}(S{\setminus } s) \\= & {} \textsf {mst}(S) + \textsf {mst}((S{\setminus } s)\cup u) - \textsf {mst}(S{\setminus } s) - \textsf {mst}(S\cup u) \end{aligned}$$

where the first inequality is due to our assumption while the second inequality is due to the induction hypothesis. Then, rearranging and adding it to (1) yields $f_{uv}(S)\le f_{uv}(S{\setminus } s)$ as desired. $\square$

## Characterization of submodularity

We are finally ready to give an efficient characterization of submodular spanning tree games.

### Theorem 1

The spanning tree game on *G* is submodular if and only if the following two conditions are satisfied.

(i) There are no violated cycles in $G_i$ for any $i<k$.
(ii) For every candidate edge *uv*, $f_{uv}({\hat{N}}(uv))\ge 0$.

Furthermore, these conditions can be verified in polynomial time.

### Proof

For necessity, assume the game is submodular. Then, Condition (*i*) is satisfied by Lemma 3 while Condition (*ii*) is satisfied trivially. For sufficiency, assume Conditions (*i*) and (*ii*) hold. Let $u,v\in N$ and $S\in {\mathcal {S}}_{uv}$. If $S\not \subseteq {\hat{N}}(uv)$, then $f_{uv}(S)=0$ by Lemma 5. On the other hand, if $S\subseteq {\hat{N}}(uv)$, then *uv* is a candidate edge. By Lemma 6,

$$\begin{aligned} f_{uv}(S)\ge f_{uv}({\hat{N}}(uv))\ge 0. \end{aligned}$$

Therefore, the game is submodular.

Checking Condition (*ii*) can clearly be done in polynomial time. Using Corollary 1, verifying Condition (*i*) reduces to searching for bad holes and bad induced diamonds in each $G_i$, which can be done efficiently. To look for bad holes, one could check if there exists a hole through a given vertex *v* for all $v\in N$ where $rv\notin E_i$. To look for bad induced diamonds, a naive implementation would involve examining all vertex subsets of size 4, which still runs in polynomial time. $\square$

## *S*-wide spanning trees

In this section, we answer another question posed in [9]. There the authors stated a necessary condition for submodularity of the spanning tree game, and left open whether their condition can be verified in polynomial time. We here show that this is indeed the case. While this is not that relevant anymore in order to characterize submodularity (since we have provided Theorem 1), it shows a nice connection with matroid intersection that might still be of interest.

### Theorem 2

(Theorem 1.2 in [9]). If the spanning tree game on *G* is submodular, then every minimum spanning tree *T* of *G* possesses the following two properties.

- It holds that $w(rv) \ge w(ru)$ for every vertex $v \in N$ and every vertex $u \in N$ on the (unique) path connecting *r* and *v* in *T*.
- For any pair of vertices $u,v \in N$ where $w(uv) < w(rv)$, the cycle obtained by adding *uv* to *T* does not contain *r*.

We show that checking Conditions (*a*) and (*b*) can be reduced to computing *S*-*wide* spanning trees, which are defined as follows. Let $G=(V,E)$ be an undirected graph with edge weights $w:E\rightarrow {\mathbb {R}}$ and a designated root $r_1\in V$. Let $S:=\left\{ s_1,\dots ,s_k\right\} \subseteq V{\setminus } r_1$ be a given set of *terminals*. We say that a spanning tree *T* of *G* is *S*-*wide* if every component of $T{\setminus } r_1$ contains at most one terminal. Equivalently, *T* is *S*-*wide* if for every $i,j\in [k]$ where $i\ne j$, the unique $s_i$-$s_j$ path on *T* contains the root $r_1$. We are interested in the following problem:

$$\begin{aligned} \textit{Given} \, (G,w,r_1,S), \, \textit{compute an} \, S\textit{-wide spanning tree of minimum weight.} \end{aligned}$$

Before solving the problem above, let us explain how one can use it to efficiently test Conditions (*a*) and (*b*) of Theorem 2. To check Condition (*a*), consider all pairs of vertices $u,v \in N$ with $w(rv) < w(ru)$, and do the following. Set $r_1 := u$ and $S := \{v,r\}$. Compute an *S*-wide spanning tree $T^*$ of minimum weight in *G*. If the weight of $T^*$ is equal to the weight of a minimum spanning tree in *G*, then $T^*$ is a minimum spanning tree of *G* violating Condition (*a*), since *u* is on the unique path from *r* to *v* in $T^*$. It is not difficult to see that this procedure will eventually find a minimum spanning tree violating Condition (*a*), if one exists.

Condition (*b*) can be checked in a similar way. Consider all ordered pairs of vertices $u,v \in N$ with $w(uv) < w(rv)$, and do the following. Set $r_1 := r$ and $S := \{u,v\}$. Compute an *S*-wide spanning tree $T^*$ of minimum weight in *G*. If the weight of $T^*$ is equal to the weight of a minimum spanning tree in *G*, then $T^*$ is a minimum spanning tree of *G* violating Condition (*b*), since adding *uv* to $T^*$ yields a cycle containing *r*. Once again, it is not difficult to see that this procedure will eventually find a minimum spanning tree violating Condition (*b*), if one exists.

We will now demonstrate how to compute an *S*-wide spanning tree of minimum weight using matroid intersection. Let $(G,w,r_1,S)$ be a given instance. Without loss of generality, we may assume that there are no edges between any pair of terminals, as every *S*-wide tree does not use them. We also assume $k\ge 2$, otherwise this reduces trivially to computing an arbitrary minimum spanning tree.

**Fig. 7:**
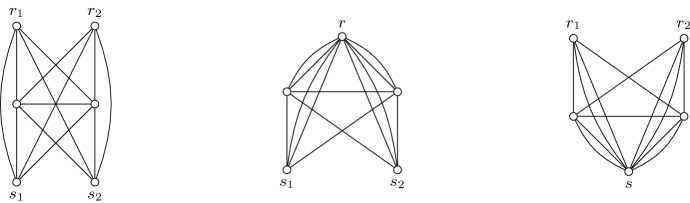
The graph $G'$ and multigraphs $G'_1, G'_2$ respectively for the input graph $K_5$ with 2 terminals

First, construct an auxiliary graph $G'=(V',E')$ from *G* as follows. Create $k-1$ copies of the root vertex $r_2,\dots ,r_k$, including its incident edges. Next, let $G'_1$ denote the multigraph obtained from $G'$ by identifying $r_1,\dots ,r_k$ into a single vertex *r*. Note that parallel edges are kept. In order to distinguish the *k* parallel edges between *r* and some vertex *v*, they will be denoted $r_iv$ for all $i\in [k]$, just like in the graph $G'$. Similarly, let $G'_2$ denote the multigraph obtained from $G'$ by identifying $s_1,\dots ,s_k$ into a single vertex *s*. See Fig. 7 for an example.

Now, consider the graphic matroids on $G'_1$ and $G'_2$, denoted $M_1 = (E',{\mathcal {I}}_1)$ and $M_2 = (E',{\mathcal {I}}_2)$ respectively. Observe that the matroids $M_1$ and $M_2$ share the same ground set $E'$ due to our notation of the parallel edges, but their independent sets are different. We would like to establish a correspondence between *S*-wide spanning trees in *G* and common bases of $M_1$ and $M_2$. Let ${\mathcal {T}}$ be the set of *S*-wide spanning trees in *G*. Moreover, let ${\mathcal {B}}_1$ and ${\mathcal {B}}_2$ be the set of bases of $M_1$ and $M_2$ respectively. Construct the function $g:2^{E'}\rightarrow 2^E$ defined by

$$\begin{aligned} g(J) := \left\{ h(e):e\in J\right\} , \end{aligned}$$

where $h:E'\rightarrow E$ is defined as

$$\begin{aligned} h(uv):= {\left\{ \begin{array}{ll} r_1v, &{}\text { if }u\in \left\{ r_1,\dots ,r_k\right\} \\ ur_1, &{}\text { if }v\in \left\{ r_1,\dots ,r_k\right\} \\ uv, &{}\text { otherwise.} \end{array}\right. } \end{aligned}$$

### Lemma 7

For every $J\in {\mathcal {B}}_1\cap {\mathcal {B}}_2$, *g*(*J*) induces an *S*-wide spanning tree in *G*.

### Proof

Let $J\in {\mathcal {B}}_1\cap {\mathcal {B}}_2$. Then, it induces a spanning tree in the multigraphs $G'_1$ and $G'_2$. Note that *g*(*J*) also induces a spanning tree in the multigraph $G'_1$. Since the input graph *G* can be obtained from the multigraph $G'_1$ by removing parallel edges, and $g(J)\subseteq E$, it follows that *g*(*J*) induces a spanning tree *T* in *G*. It is left to show that *T* is *S*-wide. For the purpose of contradiction, suppose there exist distinct indices $i,j\in [k]$ such that the unique $s_i$-$s_j$ path *P* in *T* does not contain the root $r_1$. From the definition of *h*, we know that $E(P)\subseteq J$. However, *E*(*P*) induces a non-simple walk in $G'_2$, which implies that $J\notin {\mathcal {I}}_2$. We have reached a contradiction. $\square$

### Lemma 8

For every $T\in {\mathcal {T}}$, there exists a set $J\in {\mathcal {B}}_1\cap {\mathcal {B}}_2$ such that $g(J) = E(T)$.

### Proof

Let *T* be an *S*-wide spanning tree in *G*. For every $i\in [k]$, let $r_1v_i$ be the first edge of the $r_1$-$s_i$ path on *T*. Replace each $r_1v_i$ with $r_iv_i$ and call the resulting edge set *J*. Then, *J* induces a forest *F* with *k* components in the auxiliary graph $G'$, each of which contains $r_i$ and $s_i$. Since $\delta _F(v)\ge 1$ for all $v\in V'$, *J* induces a spanning tree in the multigraph $G'_1$, as well as a spanning tree in the multigraph $G'_2$. Thus, $J\in {\mathcal {B}}_1\cap {\mathcal {B}}_2$. Moreover, $g(J) = E(T)$. $\square$

The last two lemmas imply that *g* is a surjective mapping from ${\mathcal {B}}_1\cap {\mathcal {B}}_2$ to ${\mathcal {T}}$. It is also cost-preserving because $\left| J\right| = \left| g(J)\right|$ for all $J\in {\mathcal {B}}_1$. Therefore, we can efficiently compute a minimum-weight *S*-wide spanning tree by computing a minimum-weight common basis of $M_1$ and $M_2$.
